# Supplementary material for: Ethnic and social inequalities in COVID-19 outcomes in Scotland: protocol for early pandemic evaluation and enhanced surveillance of COVID-19 (EAVE II)
Source: BMJ Open. 2021 Aug 10;11(8):e048852. doi: 10.1136/bmjopen-2021-048852 (PMC8359861; doi:10.1136/bmjopen-2021-048852)
Supplement: Author's manuscript [file bmjopen-2021-048852.draft_revisions.pdf]

BMJ Open is committed to open peer review. As part of this commitment we make the peer review history of every article we publish publicly available.

When an article is published we post the peer reviewers' comments and the authors' responses online. We also post the versions of the paper that were used during peer review. These are the versions that the peer review comments apply to.

The versions of the paper that follow are the versions that were submitted during the peer review process. They are not the versions of record or the final published versions. They should not be cited or distributed as the published version of this manuscript.

BMJ Open is an open access journal and the full, final, typeset and author-corrected version of record of the manuscript is available on our site with no access controls, subscription charges or pay-per-view fees (http://bmjopen.bmj.com).

If you have any questions on BMJ Open's open peer review process please email info.bmjopen@bmj.com

BMJ Open

Ethnic and social inequalities in COVID-19 outcomes in Scotland: protocol for early pandemic evaluation and enhanced surveillance of COVID-19 (EAVE II)

Journal:	*BMJ Open*
Manuscript ID	bmjopen-2021-048852
Article Type:	Protocol
Date Submitted by the Author:	11-Jan-2021
Complete List of Authors:	Henery, Paul; University of Glasgow, MRC/CSO Social and Public Health Sciences Unit<br>Vasileiou, Eleftheria; The University of Edinburgh, Usher Institute<br>Hainey, Kirsten ; University of Glasgow, MRC/CSO Social & Public Health Sciences Unit<br>Buchanan, Duncan; Public Health Scotland<br>Harrison, Ewen ; University of Edinburgh<br>Leyland, Alastair; MRC/CSO Social and Public Health Sciences Unit, University of Glasgow<br>Alexis, Thomas; Scottish Government<br>Robertson, Chris; University of Strathclyde, Department of Mathematics and Statistics<br>Agrawal, Utkarsh; University of St Andrews, School of Medicine<br>Ritchie, Lewis; Aberdeen University, General Practice and Primary Care<br>Stock, Sarah; The University of Edinburgh Usher Institute of Population Health Sciences and Informatics, Centre for Medical Informatics; Public Health Scotland,<br>McCowan, Colin ; University of St. Andrews<br>Docherty, Annemarie; University of Edinburgh, Anaesthesia and Critical Care<br>Kerr, Steven; University of Edinburgh<br>Marple, James; University of Edinburgh<br>Wood, Rachael; NHS National Services Scotland, Information Services Division; University of Edinburgh<br>Moore, Emily; Public Health Scotland<br>Simpson, Colin; Victoria University of Wellington; University of Edinburgh<br>Sheikh, Aziz; University of Edinburgh, Division of Community Health Sciences<br>Katikireddi, Srinivasa; University of Glasgow
Keywords:	COVID-19, EPIDEMIOLOGY, Protocols & guidelines < HEALTH SERVICES ADMINISTRATION & MANAGEMENT, Public health < INFECTIOUS DISEASES

I, the Submitting Author has the right to grant and does grant on behalf of all authors of the Work (as defined in the below author licence), an exclusive licence and/or a non-exclusive licence for contributions from authors who are: i) UK Crown employees; ii) where BMJ has agreed a CC-BY licence shall apply, and/or iii) in accordance with the terms applicable for US Federal Government officers or employees acting as part of their official duties; on a worldwide, perpetual, irrevocable, royalty-free basis to BMJ Publishing Group Ltd ("BMJ") its licensees and where the relevant Journal is co-owned by BMJ to the co-owners of the Journal, to publish the Work in this journal and any other BMJ products and to exploit all rights, as set out in our licence.

The Submitting Author accepts and understands that any supply made under these terms is made by BMJ to the Submitting Author unless you are acting as an employee on behalf of your employer or a postgraduate student of an affiliated institution which is paying any applicable article publishing charge ("APC") for Open Access articles. Where the Submitting Author wishes to make the Work available on an Open Access basis (and intends to pay the relevant APC), the terms of reuse of such Open Access shall be governed by a Creative Commons licence – details of these licences and which Creative Commons licence will apply to this Work are set out in our licence referred to above.

Other than as permitted in any relevant BMJ Author's Self Archiving Policies, I confirm this Work has not been accepted for publication elsewhere, is not being considered for publication elsewhere and does not duplicate material already published. I confirm all authors consent to publication of this Work and authorise the granting of this licence.

For peer review only

Ethnic and social inequalities in COVID-19 outcomes in Scotland: protocol for early pandemic evaluation and enhanced surveillance of COVID-19 (EAVE II)

Paul M. Henery [1]
Eleftheria Vasileiou [2]
Kirsten J. Hainey [1]
Duncan Buchanan [3]
Ewen M. Harrison [2]
Alastair H. Leyland [1]
Thomas Alexis [4]
Chris Robertson [5]
Utkarsh Agrawal [6]
Lewis D. Ritchie [7]
Sarah J. Stock [2]
Colin McCowan [6]
Annemarie Docherty [2]
Steven Kerr [2]
James Marple [2]
Rachael Wood [3, 2]
Emily Moore [3]
Colin R. Simpson [8]
Aziz Sheikh [2]
S. Vittal Katikireddi [1, 3]

[1] MRC/CSO Social & Public Health Sciences Unit, University of Glasgow, UK
[2] University of Edinburgh, UK
[3] Public Health Scotland, UK
[4] Scottish Government, UK
[5] University of Strathclyde, UK
[6] University of St Andrews, UK
[7] University of Aberdeen, UK
[8] Victoria University of Wellington, New Zealand

Corresponding author:
Srinivasa Vittal Katikireddi
vittal.katikireddi@glasgow.ac.uk

MRC/CSO Social and Public Health Sciences Unit
University of Glasgow
Berkeley Square
99 Berkeley Street
Glasgow
G3 7HR

Word count: 3,821

For peer review only

ABSTRACT

Introduction: Evidence from previous pandemics, as well as the current COVID-19 pandemic, has found that risk of infection and severity of disease is disproportionately higher for ethnic minority groups, and those in lower socio-economic positions. It is imperative that interventions to prevent the spread of COVID-19 are targeted towards high-risk populations. We will investigate the associations between social characteristics (such as ethnicity, occupation and socio-economic position) and COVID-19 outcomes and the extent to which known characteristics and risk factors might explain any observed relationships in Scotland.
The primary objectives of this study are to: a) describe the epidemiology of COVID-19 by social factors; b) examine receipt of treatment and prevention of COVID-19 by social factors; c) quantify ethnic/social differences in adverse COVID-19 outcomes; d) explore potential mediators of relationships between social factors and SARS-CoV-2 infection and COVID-19 prognosis; and e) examine whether occupational COVID-19 differences differ by other social factors.

Methods and analysis: We will use a national cohort comprising the adult population of Scotland who completed the 2011 Census and were living in Scotland on March 31st 2020 (approximately 4.3 million). Census data will be linked to the EAVE II cohort consisting of primary care, secondary care, laboratory data and death records. Sensitivity/specificity and positive/negative predictive values will be used to assess ethnicity coding quality. Descriptive statistics will be used to examine differences in treatment and prevention of COVID-19. Poisson/Cox regression analyses, and mediation techniques will examine ethnic and social differences, and drivers of inequalities in COVID-19. Effect modification (on additive and multiplicative scales) between key variables (such as ethnicity and occupation) will be assessed.

Ethics and dissemination: Ethical approval was obtained from the National Research Ethics Committee, South East Scotland 02. We will present findings of this study at international conferences, in peer-reviewed journals and to policymakers.

STRENGTHS AND LIMITATIONS OF THIS STUDY

- This study uses a near-complete cohort of the Scottish population, providing considerable statistical power to investigate social inequalities in COVID-19.
- Linkage of the Scottish Census to administrative data allows for evaluation of differences in COVID-19 across specific occupational classes, which is not available in any other dataset in Scotland.
- Given the Census data are from 2011 and are being linked to data from 2020-21, some occupational and social data may be out-of-date
- Individuals who moved to Scotland post-Census will not be included in the cohort, for the same reasons as above

For peer review only

INTRODUCTION

Historically, pandemics have disproportionately affected specific groups within the population. For example, during the Spanish influenza pandemic of 1918, individuals from poorer socio-economic positions experienced higher incidence of influenza in the United States.[1] These findings have been replicated in various international settings using data from the 1918 pandemic.[2] Similar patterns were observed following the H1N1 influenza pandemic in 2009 in Canada, with an increased risk of hospitalisation amongst individuals with lower educational attainment or resident in areas of greater deprivation.[3] English data demonstrated a socio-economic gradient in mortality during the 2009 pandemic, with the highest risk of death observed in those living in deprived areas.[4 5] Ethnicity was also identified as a risk factor for mortality during the 2009 pandemic, with those in non-White ethnic groups at increased risk of mortality from H1N1 influenza compared to White populations in the UK.[5] The mechanism underlying these socio-economic patterns has been hypothesised as resulting from a combination of different exposure and susceptibility to infection, and differing access to healthcare services.[6] Understanding the role of ethnic, socio-economic and occupational risk factors in both risk and outcome of infection during a pandemic is essential for health service planning, targeting preventative measures and informing future modelling efforts.

The Severe Acute Respiratory Syndrome coronavirus-2 (SARS-CoV-2) and its resulting disease (COVID-19) is spreading rapidly worldwide, causing a high burden of mortality and morbidity.[7] The differential risks of infection in ethnic minority groups have been examined during the COVID-19 pandemic. Epidemiological analyses within England have found higher risks among some minority ethnic groups, both for risk of infection and for the consequences of infection.[8-12] These findings have been supported by international evidence, which suggests individuals from Black and South Asian ethnic groups are at increased risk of experiencing COVID-19 and its resultant harms.[11 13]

Emerging evidence suggests that marked socio-economic inequalities in risk and outcome of COVID-19 exist, yet empirical evidence describing the role of socio-economic factors in Scotland is limited.[10] Initial analyses in Scotland have been subject to considerable misclassification of ethnicity (due to systematically incomplete data within health records) and limited by using a broad three-category classification of ethnicity in a small case-control sample;[14] however, survey-based research with more specific ethnicity classifications has suggested that ethnic minorities may be more hesitant to be vaccinated against COVID-19.[15] Analyses have therefore shown inconsistent findings. Understanding how sociodemographic factors are related to infection risk and prognosis, as well as monitoring inequalities by area-level (such as indices of multiple deprivations) and individual/household-level (such as social class or household tenure) socio-economic position, is essential for health service planning, targeting prevention efforts and informing future modelling efforts. In addition, our understanding of why inequalities in COVID-19 outcomes exist remain limited. For example, differences in occupation, household composition and healthcare could operate as important mediating mechanisms, which could potentially be targeted to mitigate ethnic inequalities in COVID-19.

The Early Assessment of Vaccine and Anti-Viral Effectiveness II (EAVE II) study[16-18] is an ongoing project which tracks the epidemiology of COVID-19 in Scotland in real-time, and aims to evaluate the effectiveness of future vaccinations in reducing spread of the infection. Whilst the main EAVE II project will stratify COVID-19 rates and vaccine effectiveness by demographic characteristics such as age and region, it will not examine the role of ethnicity and social factors in detail. Linkage of the EAVE II dataset to other administrative sets, in particular the 2011 Scotland Census, allows the assessment of the role of social and ethnic inequalities on risk of SARS-CoV-2 infection and subsequent disease outcomes. This provides better understanding of groups at higher risk from COVID-19 within the Scottish population compared to administrative data sources (which typically

For peer review only - http://bmjopen.bmj.com/site/about/guidelines.xhtml

lack detailed information about social characteristics), yielding valuable information for clinicians and healthcare planners as the pandemic continues to evolve. Learning from this pandemic may also yield longer term lessons for public health policy.

Aim and objectives

This EAVE II sub-study aims to quantify social inequalities in COVID-19 related outcomes in Scotland using linked Census and administrative data, as well as examining the mechanisms through which inequalities in COVID-19 arise. Our specific primary objectives are as follows:

a. To describe the epidemiology of COVID-19 by: i) ethnicity; ii) socio-economic position; iii) occupation; and iv) residential circumstances
b. To monitor receipt of COVID-related treatment and preventive interventions across social and ethnic groups
c. To quantify ethnic and social differences in the risk of adverse outcomes (hospital admission, critical care admission and mortality) from COVID-19
d. To explore the potential contribution of pre-existing health conditions and risk factors to ethnic and social inequalities in COVID-19 and prognosis
e. To investigate if occupational (and potentially socio-economic or ethnic) differences in COVID-19 risks are modified by other social factors.

In addition, we have one secondary objective:

a. To assess the quality of ethnicity coding within NHS administrative datasets

Objective c will be examined for three groups: (i) laboratory confirmed SARS-CoV-2 infection; (ii) clinically diagnosed SARS-CoV-2 infection; (iii) serological evidence of previous infection with SARS-CoV-2.

METHODS AND ANALYSIS

Study design and population

We will perform analyses on a national prospective cohort of the Scottish population, including all individuals who meet the following criteria: a) alive on the 1st March 2020; b) resident in Scotland on that date; and c) present in the 2011 Census in Scotland. A start date of 1st March 2020 has been selected as the first case of laboratory confirmed SARS-CoV-2 in Scotland was identified on 2nd March 2020.[19] Analyses will focus on the adult population aged 16 years and over, with some analyses limited to older age groups (e.g. aged 25+ years on the Census 2011 date when analysing highest education level).

Databases

The Community Health Index (CHI) is a unique numerical identifier, assigned to every individual registered to receive healthcare in Scotland, which can be used to identify and link all healthcare interactions and records for an individual.[20] Linkage of these administrative datasets with the 2011 Census data will allow the examination of clinically diagnosed and laboratory confirmed SARS-CoV-2 infection, deaths and potential confounders (Fig. 1). The CHI is mandatory for anyone receiving medical treatment, but individuals who are not linked to the Census will be excluded from the cohort.[20] Furthermore, individuals known to have emigrated (through GP de-registration) prior to

For peer review only

the pandemic will be excluded. Data linkage and analyses will take place within the National Safe Haven, a secure and trusted research environment (TRE).[21]

Census

In Scotland, the national Census is conducted every 10 years by means of a mandatory survey sent to every household in the country.[22] The most recent Census was conducted in 2011 and forms the population spine of our cohort. The Census extract for this study contains demographic information including ethnic group, religion and country of birth, marital status, socio-economic position (SEP) indicators such as highest educational attainment, employment status and occupation, household information including persons per room, tenure and number of cars, and variables on pre-pandemic health status identifying long-term illnesses and disability status. Census data are necessary to identify our exposures of interest, as well as to study potential mediating pathways and effect modification. The necessary information required from the Census (e.g. ethnicity, occupation) is not reliably available from other sources. While ethnicity is recorded in some health datasets in Scotland, it is often missing, reported inconsistently across datasets, and not always based on self-report, which is considered the gold standard for establishing ethnicity.[23]

Laboratory data

To identify individuals with confirmed SARS-CoV-2 infection, positive viral reverse transcriptase polymerase chain reaction (RT-PCR) results will be confirmed using Electronic Communication of Surveillance in Scotland (ECOSS) data, a national repository of diagnostic and reference laboratory data on testing for viral infections including COVID-19.[24] To identify individuals with serologically confirmed infection, serology data will be provided via the Enhanced Surveillance of COVID-19 in Scotland (ESoCiS) programme undertaken by Public Health Scotland (PHS) on behalf of the Scottish Government.[25]

Secondary care

The Scottish Morbidity Record (SMR), maintained by PHS, is the primary repository for data describing hospital admissions in Scotland.[26] We will use data from SMR01, which details general inpatient admissions, and SMR02, which details obstetric admissions. We will include data from the Rapid Preliminary Inpatient Data (RAPID) dataset. The RAPID data are collected quickly, in an unprocessed state and are therefore available more quickly than SMR data,[27] which is necessary given the pertinent nature of our study. Data about intensive care unit admissions in adults will be included by linking data from the Scottish Intensive Care Society Audit Group (SICSAG).[28] These datasets use International Classification of Diseases (ICD-10) codes to record an individual's diagnoses. ICD-10 codes will be used to identify a clinical diagnosis of COVID-19 in the absence of a positive test result for those admitted to hospital with diagnosed COVID-19. Scottish Ambulance Service (SAS) and Accident and Emergency (A&E) records, also provided by PHS, will be used to identify people with clinically suspected COVID-19.

The Prescribing Information System (PIS) is the repository for all prescriptions requested for individuals by general practices,[29] and the Hospital Electronic Prescribing and Medicines Administration (HEPMA) is the equivalent for prescribing and administration of medicine in a hospital setting.[30] Both of these datasets use the British National Formulary (BNF), a standardised medication classification system.[31] We will use BNF codes to identify prescribed drugs or administered medicine used to treat COVID-19, identify comorbidities (such as diabetes and hypertension) and allow the study of longer-term consequences of COVID-19.

For peer review only

Primary care

Just under 100% of the population of Scotland is registered with a general practice (GP), which provides free-at-point-of-delivery primary healthcare services.[32] NHS 24 is the Scottish national telephone service for the general public, which provides clinical triage and advice for those experiencing non-critical ill health during the out-of-hours period. This was expanded to provide advice to individuals with suspected COVID-19 at any time during the pandemic.[33] We will use GP consultation data and NHS24 out-of-hours data to identify clinically suspected COVID-19. In addition, data from the recently established COVID-19 clinical assessment centres will also be included for this purpose.

Deaths

National Records for Scotland (NRS) provides data about deaths attributed to COVID-19, which will be included in our dataset.[34] ICD-10 codes will be used to identify deaths occurring in individuals with clinically confirmed COVID-19 within 28 days of death (U07.1, U07.2).

Exposures and covariates

We will examine four categories of social factors in relation to the study's primary outcomes: ethnicity, socio-economic position (SEP), occupation and residential circumstances. Ethnicity will be categorised based on standard Census classifications,[35] self-reported in sixteen groups. We will try to analyse ethnicity using as detailed categorisations as possible, subject to having adequate numbers for meaningful statistical analysis. SEP will be classified at both area-level (the Scottish Index of Multiple Deprivation [SIMD]) and individual level (e.g. education level and housing tenure). The SIMD is a measure of the relative deprivation of broadly heterogeneous population-level "data zones" within Scotland, classifying deprivation using the following weighted domains: employment, income, health, crime, housing, education and access to services.[36] The SIMD is typically operationalised using either quintiles or deciles, with quintile/decile 1 referring to the most deprived 20% or 10% of the population, respectively. Marital status will consist of five categories as per the Census: single (never married), married, separated, divorced and widowed.[37] Housing tenure will consist of seven categories as per the Census: owned (outright), owned (mortgage), social rented (council), social rented (other), private rented (landlord/letting agency), private rented (other/rent-free), or communal.[38] Occupation will be measured using the Standard Occupational Classification 2000, consisting of major, sub-major, minor and unit group levels.[39] Employment status, weekly hours worked, and type of industry (coded using Standardised Industrial Classification, SIC) will be used to explore potential differential workplace exposure. Residential circumstances will be classified using the following parameters: housing tenure, persons per room, household size and age range of household (difference between oldest and youngest permanent occupant).

The following additional characteristics will be used to address confounding, explore mediation and for assessing effect modification. Cultural factors will be investigated, including religion, and English language ability via a four-point Likert scale (very well, well, not well, not at all). Geographical variables will include 32 local authorities (LA) comprising local government in Scotland. Whilst these LA have some devolved powers, primarily in service provision,[40] the most pertinent local factor for this study is that COVID-19 mitigation levels are, at the time of writing, allocated by a LA-level tiering system.[41] We will explore the role of rurality using the Scottish Government Urban Rural Classification (URC), which can be used as a simple two-fold measure (defining "rurality" as areas with a population of <3,000) at its simplest, or an eight-fold measure at its most complex).[42] Pre-pandemic health status will be assessed on the basis of general self-reported health, captured using a five-point Likert scale (very good, good, fair, bad, very bad), and the presence of the following

For peer review only

additional self-reported health circumstances as recorded in the Census: long-term condition, mental health condition, other condition, hearing impairment, visually impairment, learning disability, developmental disorder, and physical disability. Behavioural risk factors (such as smoking status and body mass index) and health conditions (including pregnancy) will also be identified from GP and hospital data.

Outcome definitions

The primary outcomes of this study will be laboratory confirmed SARS-CoV-2 infection, clinically diagnosed COVID-19, and serologically confirmed evidence of previous SARS-CoV-2 infection. Laboratory confirmed SARS-CoV-2 infection will be defined as positive RT-PCR via the ECOSS dataset. Serologically confirmed SARS-CoV-2 infection will be defined as positive antibodies on serology testing as identified through ESoCiS data, reflecting immunological evidence of previous infection. Clinically diagnosed COVID-19 disease will be defined as the requisite ICD-10 code being recorded on discharge from hospital via SMR, intensive care via SICSAG, or on NRS death records. Clinically suspected COVID-19 disease will be used as a secondary outcome in the absence of laboratory confirmed COVID-19 and recorded via SAS or A&E attendance records, GP consultation or out-of-hours records, COVID-19 clinical assessment centre data, or NHS 24 logs. Given testing practices are changing over time, we will conduct sensitivity analyses by stratifying analyses by time period and consider weighting analyses to account for differential testing practices.

Secondary outcomes include uptake of vaccination for COVID-19, as recorded in GP records or the vaccine delivery app, and being prescribed treatment for COVID-19 either via the PIS or HEPMA. We will also examine adverse outcomes following COVID-19 infection: admission to hospital, critical care admission, or death. Hospital admission or death will be defined on the basis of either COVID-19 (laboratory confirmed or clinically diagnosed) recorded on a hospital admission in SMR01 or death record (using ICD10 codes U07.1, U07.2,) or positive test, or admission in RAPID within 14 days of a SARS-CoV-2 RT-PCR positive test.[43] Critical care admissions will be defined on the basis of presence on the SICSAG database and a relevant hospital admission (using ICD10 codes U07.1, U07.2), or positive test.

Statistical analysis

Epidemiology of COVID-19 by ethnicity and social factors

We will describe the epidemiology of COVID-19 by ethnic and social factors, specifically in relation to: a) incidence of confirmed SARS-CoV-2 infection; b) incidence of clinically diagnosed disease; and c) evidence of previous infection. We will use summary statistics to describe the cohort by presence and absence of confirmed, diagnosed and suspected COVID-19. We will perform a descriptive analysis of the number of cases by exposure groups in the first instance, to determine the level of disaggregation feasible. In addition to analysing inequalities by ethnicity and social factors, we will also investigate the inequalities by country of birth (i.e. migration-related inequalities).
Poisson regression with robust standard errors will be used to estimate risk ratios for the risk of infection across exposure groups. We will consider age and sex to be key potential confounders for all analyses, with adjustment for local authority also planned for most analyses (to account for the differential spread of the infection across geographies, and local mitigation levels). If case numbers allow, we will examine the incidence of confirmed and suspected COVID-19 by sex (and for ethnicity, country of birth). We can assess whether findings are robust to more stringent definitions of confirmed and suspected infection, e.g. by restricting the definition of a confirmed case to one with positive viral RT-PCR, and restricting the definition of a suspected case to exclude people with a negative viral RT-PCR result.

For peer review only

Surveillance of preventative treatment and interventions for COVID-19 across social and ethnic factors

We will describe treatment for and prevention of COVID-19 by ethnicity and other social factors (e.g. socio-economic position, occupation), especially in relation to vaccination uptake. We will tabulate those who have been vaccinated as a proportion of the total eligible in different social subgroups, from when the vaccine is available and delivered nationally. This will be monitored over time to investigate whether there is a change in vaccination rates for different subgroups. If we have sufficient numbers of people vaccinated, we will also stratify these analyses by demographic factors. Similarly, we will also investigate the potential ethnic and other social differences in treatment for COVID-19 during acute infection and post-COVID via GP prescribing or hospital-administered medical care.

Inequalities in risk of adverse health outcomes from COVID-19

We will quantify ethnic and social inequalities in the risk of adverse outcomes (hospital admission, critical care admission and mortality) from COVID-19. We will perform a descriptive analysis of the number of cases by exposure group in the first instance, to help determine the level of disaggregation feasible. We will follow a previously published approach[44] to analyse COVID-19 survival outcomes using Cox proportional hazards models from the date of a positive test result (or first date of diagnosis). We will conduct sensitivity analyses excluding people resident in care homes at the study start date. We will consider Fine & Gray competing risks models (to account for non-COVID-19 deaths) for sensitivity analysis[45] or alternatively by applying inverse probability weights to address competing risks.

Mediation analyses to explore drivers of inequalities in COVID-19

We will explore the contribution of several potential mediators of ethnic and social inequalities in COVID-19. The extent of these analyses will be determined by the magnitude of any observed inequalities in COVID-19 outcomes. Potential mediators to be investigated for ethnic inequalities include occupation, socio-economic position, cultural factors (such as language ability and country of birth) and pre-pandemic health (assessed through census, GP records and hospitalisations data). We will conduct cross-tabulations of the relationships between exposures, potential mediators and outcomes, followed by a set of nested regression models for the same purpose. Following this, we will pursue more formal mediation analyses. We expect to use G methods,[46] such as marginal structural modelling, to estimate the direct and indirect effect of ethnicity mediated through the mediating factors. We will investigate the extent to which inequalities might arise from differential exposure compared to differential susceptibility.[47] Our analysis will be informed by a Directed Acyclic Graph (DAG) (Fig. 2). Given we are uncertain about whether the country of birth and geography are confounders or mediators, we will conduct sensitivity analyses without adjusting for these variables. In addition to analysing inequalities by ethnicity, we will investigate inequalities by country of birth to explore migration-related inequalities. We will consider conducting similar mediation analyses for socio-economic inequalities in COVID-19, depending on the extent of inequalities observed.

Effect modification

We will investigate whether interactions exist for key combinations of exposure variables, such as ethnicity, socio-economic position and occupation. We will study whether occupational risks of COVID-19 differ by ethnic group. Occupation will be assessed on the basis of Standard Occupational Classification 2000 codes, aggregated from three-digit codes to a level that meets disclosure control

For peer review only

requirements. We will perform descriptive analysis of the number of cases by occupation initially, followed by cross-tabulation by ethnicity. We will then estimate relative risks for outcomes of interest and check for evidence of effect modification on an additive and multiplicative scale. Should we observe evidence that occupational risk differs by ethnicity, we will calculate relative excess risks due to interaction.[48] Similar analyses will be conducted to assess potential effect modification for key combinations of exposure groups, as prioritised by our policy partners.

Assessment of quality of ethnicity coding

We will assess the quality of ethnicity coding within NHS administrative datasets, in order to assess whether, in the absence of Census data, administrative data can be reliably used to study ethnic inequalities. We will start by assessing the proportion of information on ethnicity missing within each dataset. Following this, we will consider the 2011 Census to be our 'gold standard' dataset for comparison purposes. We will quantify the prevalence of different ethnic groups within each dataset and assess the sensitivity, specificity, positive predictive value and negative predictive value, following the methods used in previous validation studies.[49] If numbers of cases allow, we will stratify analyses by broad age group, sex and country of birth.

Sample size

Accounting for non-linkage and non-participation in the Census (via migration or birth after the Census or otherwise), we estimate that the size of the cohort will be 4.25 million people. We are confident based on research from previous pandemics in similar-sized populations[50] that there will be sufficient statistical power to perform our planned analyses. For example, for the African ethnic group which is one of the least frequent ethnic groups in Scotland, considering COVID-19 deaths (the least common outcome), we anticipate there being approximately 30 COVID-19 deaths from the first wave in the African ethnic group which exceeds the number of events in previous completed analyses. Utlimately, the total number of events is dependent on the trajectory of the COVID-19 pandemic in Scotland, and statistical power will be assessed on an outcome-by-outcome basis.

Patient and public involvement (PPI)

An EAVE II PPI group has been established, which includes diverse ethnic and socio-economic representation. Study plans have been discussed with the group and this has confirmed the importance of investigating a broad range of social factors (including socio-economic position, as well as ethnicity). The study design was initially developed by the study investigators without PPI due to delays in being able to establish the PPI group, but more substantive input into the creation of detailed analysis plans is now ongoing. Findings will be discussed regularly with the group and revised on the basis of their input accordingly. The PPI group will also inform the development of dissemination plans.

ETHICS AND DISSEMINATION

This study, as part of the wider EAVE II project, has been approved by the National Research Ethics Committee, South East Scotland 02. The study has also received approval from the Public Benefit and Privacy Panel for Health and Social Care (2021-0015), and approval from the Statistics Public Benefit and Privacy Panel (2021-0115). Findings from this study will be published in peer-reviewed journals and presented at international conferences. Analyses will be presented to policymakers within UK and Scottish governments and to relevant public health practitioners in Scotland throughout the course of the study. STROBE and RECORD guidelines will be used in reporting the findings of the study.

For peer review only

AUTHORS' CONTRIBUTIONS

SVK designed the study. PMH, EV, DB, KJH and CRS critically revised the study design. PMH drafted the manuscript. EV, KJH, DB, EMH, AHL, TA, CR, UA, LDR, SJS, CM, AD, SK, RW, EM, AS and SVK critically revised the manuscript. SVK obtained funding for the study.

FUNDING STATEMENT

PMH, SVK, AHL and KH are funded by the Medical Research Council (MC_UU_12017/13) and Scottish Government Chief Scientist Office (SPHSU13). SVK is funded by an NRS Senior Clinical Fellowship (SCAF/15/02). EAVE II is funded by the Medical Research Council (MR/R008345/1) with the support of BREATHE - The Health Data Research Hub for Respiratory Health [MC_PC_19004], which is funded through the UK Research and Innovation Industrial Strategy Challenge Fund and delivered through Health Data Research UK; with additional support from the Scottish Government DG Health and Social Care.

COMPETING INTERESTS STATEMENT

SVK is co-chair of the Scottish Government's Expert Reference Group on ethnicity and COVID-19 and a member of the UK Government's Scientific Advisory Group on Emergencies (SAGE) subgroup on ethnicity and COVID-19. AS is a member of the Scottish Government's Chief Medical Officer's COVID-19 Advisory Group, the Scottish Government's Expert Reference Group on ethnicity and COVID-19 and the UK Government's Subgroup on Risk Stratification of the New and Emerging Respiratory Virus Threats Group (NERVTAG). LR acts as a medical advisor to Scottish Government on primary care and public health, including vaccination issues. CRS reports grants from the UK National Institute for Health Research, Medical Research Council and New Zealand Health Research Council, and The Ministry of Business, Innovation and Employment.

FIGURE LEGENDS

Fig. 1: Data flow diagram for linkage of census and administrative data comprising cohort
Blue box: Census data
Yellow box: Administrative data
Green box: Linked data
Dark background: Data processing

Fig. 2: Directed Acyclic Graph (DAG) of the causal pathways between ethnicity and COVID-19
Yellow box: Outcome
Blue box: Exposure
Green box: Confounder
White box with green outline: Confounder (unmeasured)
White box with blue outline: Mediator
Blue arrow: Main effect
Green arrow: Confounding
Light green arrow: Unmeasured confounding
Light blue arrow: Mediated effect

REFERENCES

For peer review only

1. Sydenstricker E. The incidence of influenza among persons of different economic status during the epidemic of 1918. *Public Health Reports (1896-1970)* 1931;46(4):154-70.
2. Bambra C, Riordan R, Ford J, et al. The COVID-19 pandemic and health inequalities. *J Epidemiol Community Health* 2020;74(11):964-68. doi: 10.1136/jech-2020-214401 [published Online First: 2020/06/15]
3. Lowcock EC, Rosella LC, Foisy J, et al. The social determinants of health and pandemic H1N1 2009 influenza severity. *Am J Public Health* 2012;102(8):e51-8. doi: 10.2105/AJPH.2012.300814 [published Online First: 2012/06/16]
4. Rutter PD, Mytton OT, Mak M, et al. Socio-economic disparities in mortality due to pandemic influenza in England. *Int J Public Health* 2012;57(4):745-50. doi: 10.1007/s00038-012-0337-1 [published Online First: 2012/02/03]
5. Zhao H, Harris RJ, Ellis J, et al. Ethnicity, deprivation and mortality due to 2009 pandemic influenza A(H1N1) in England during the 2009/2010 pandemic and the first post-pandemic season. *Epidemiol Infect* 2015;143(16):3375-83. doi: 10.1017/S0950268815000576 [published Online First: 2015/04/09]
6. P. B, A. R, S. E, et al. Pandemic influenza planning in the United States from a health disparities perspective. *Emerg Infect Dis* 2008;14(5):709-15.
7. World Health Organization. Coronavirus disease 2019 (COVID-19): situation report 91, 2020.
8. Aldridge RW, Lewer D, Katikireddi SV, et al. Black, Asian and Minority Ethnic groups in England are at increased risk of death from COVID-19: indirect standardisation of NHS mortality data. *Wellcome Open Res* 2020;5:88. doi: 10.12688/wellcomeopenres.15922.2 [published Online First: 2020/07/10]
9. Intensive Care National Audit & Research Centre. ICNARC report on COVID-19 in critical care: England, Wales and Northern Ireland. London, 2020.
10. Niedzwiedz CL, O'Donnell CA, Jani BD, et al. Ethnic and socioeconomic differences in SARS-CoV-2 infection: prospective cohort study using UK Biobank. *BMC Med* 2020;18(1):160. doi: 10.1186/s12916-020-01640-8 [published Online First: 2020/05/30]
11. White C, Nafilyan V. Coronavirus (COVID-19) related deaths by ethnic group, England and Wales: 2 March 2020 to 10 April 2020. In: Statistics OfN, ed., 2020.
12. Williamson E, Walker AJ, Bhaskaran K, et al. OpenSAFELY: factors associated with COVID-19-related hospital death in the linked electronic health records of 17 million adult NHS patients. *Preprint* 2020 doi: 10.1101/2020.05.06.20092999
13. Sze S, Pan D, Nevill CR, et al. Ethnicity and clinical outcomes in COVID-19: A systematic review and meta-analysis. *EClinicalMedicine* 2020;29:100630. doi: 10.1016/j.eclinm.2020.100630 [published Online First: 2020/11/18]
14. McKeigue PM, Weir A, Bishop J, et al. Rapid Epidemiological Analysis of Comorbidities and Treatments as risk factors for COVID-19 in Scotland (REACT-SCOT): A population-based case-control study. *PLoS Med* 2020;17(10):e1003374. doi: 10.1371/journal.pmed.1003374 [published Online First: 2020/10/21]
15. Robertson E, Reeve KS, Niedzwiedz CL, et al. Predictors of COVID-19 vaccine hesitancy in the UK Household Longitudinal Study. *Preprint* 2021 doi: 10.1101/2020.12.27.20248899
16. Simpson CR, Beever D, Challen K, et al. The UK's pandemic influenza research portfolio: a model for future research on emerging infections. *The Lancet Infectious Diseases* 2019;19(8):e295-e300. doi: 10.1016/s1473-3099(18)30786-2
17. Simpson CR, Robertson C, Vasileiou E, et al. Early Pandemic Evaluation and Enhanced Surveillance of COVID-19 (EAVE II): protocol for an observational study using linked Scottish national data. *BMJ Open* 2020;10(6):e039097. doi: 10.1136/bmjopen-2020-039097 [published Online First: 2020/06/23]
18. Simpson CR, Thomas BD, Challen K, et al. The UK hibernated pandemic influenza research portfolio: triggered for COVID-19. *The Lancet Infectious Diseases* 2020;20(7) doi: 10.1016/s1473-3099(20)30194-8

For peer review only

19. Filipe AdS, Shepherd J, Williams T, et al. Genomic epidemiology of SARS-CoV-2 spread in Scotland highlights the role of European travel in COVID-19 emergence. *Preprint* 2020 doi: 10.1101/2020.06.08.20124834
20. ISD Scotland. ISD Scotland data dictionary: CHI number [Available from: https://www.ndc.scot.nhs.uk/Dictionary-A-Z/Definitions/index.asp?Search=C&ID=128&Title=CHI%20Number accessed 5 Jan 2021.
21. NHS Scotland. Data Safe Haven 2021 [Available from: https://www.nhsresearchscotland.org.uk/research-in-scotland/data/safe-havens accessed 5 Jan 2021.
22. National Records of Scotland. What is Scotland's Census? 2018 [Available from: https://www.scotlandscensus.gov.uk/what-census accessed 5 Jan 2021.
23. NHS National Services Scotland. Measuring use of health services by equality group. NSS Information and Intelligence. Edinburgh, 2017.
24. Health Protection Scotland. Data and surveillance 2020 [Available from: https://www.hps.scot.nhs.uk/data/ accessed 5 Jan 2021.
25. Health Protection Scotland. Enhanced surveillance of coronavirus (COVID-19) 2020 [Available from: https://www.hps.scot.nhs.uk/a-to-z-of-topics/enhanced-surveillance-of-coronavirus-covid-19/ accessed 5 Jan 2021.
26. ISD Scotland. SMR datasets: episode management: SMR record type [Available from: https://www.ndc.scot.nhs.uk/Data-Dictionary/SMR-Datasets//Episode-Management/SMR-Record-Type/ accessed 5 Jan 2021.
27. Public Health Scotland. System Watch 2020 [Available from: https://www.isdscotland.org/Products-and-Services/System-Watch/ accessed 5 Jan 2021.
28. Scottish Intensive Care Society Audit Group. About SICSAG [Available from: https://www.sicsag.scot.nhs.uk/about/main.html accessed 5 Jan 2021.
29. ISD Scotland. National data catalogue: national datasets [Available from: https://www.ndc.scot.nhs.uk/National-Datasets/data.asp?SubID=9 accessed 5 Jan 2021.
30. eHealth. HEPMA 2015 [Available from: https://www.ehealth.scot/case-studies/hepma/ accessed 5 Jan 2021.
31. BNF Publications. Our organisation 2021 [Available from: https://www.bnf.org/about/ accessed 5th Jan 2021.
32. A. B, R. T, J. M. NHS number and the systems used to manage them: an overview for research users. In: CLOSER, ed. Resource Report. London, 2018.
33. NHS 24. When to phone 111 2019 [Available from: https://www.nhs24.scot/111/when-to-phone-111/ accessed 5 Jan 2021.
34. National Records of Scotland. Deaths involving coronavirus (COVID-19) in Scotland 2021 [Available from: https://www.nrscotland.gov.uk/statistics-and-data/statistics/statistics-by-theme/vital-events/general-publications/weekly-and-monthly-data-on-births-and-deaths/deaths-involving-coronavirus-covid-19-in-scotland accessed 5 Jan 2021.
35. National Records of Scotland. Ethnic group 2018 [Available from: https://www.scotlandscensus.gov.uk/variables-classification/ethnic-group accessed 5 Jan 2021.
36. The Scottish Government. Scottish Index of Multiple Deprivation 2020 2020 [Available from: https://www.gov.scot/collections/scottish-index-of-multiple-deprivation-2020/ accessed 5 Jan 2021.
37. National Records of Scotland. Marital status 2018 [Available from: https://www.scotlandscensus.gov.uk/marital-status-0 accessed 5 Jan 2021.
38. National Records of Scotland. Tenure of household 2018 [Available from: https://www.scotlandscensus.gov.uk/tenure-household accessed 5 Jan 2021.

For peer review only

39. Office for National Statistics. SOC 2000 [Available from: https://www.ons.gov.uk/methodology/classificationsandstandards/standardoccupationalclassificationsoc/socarchive accessed 5 Jan 2021.
40. The Scottish Government. Local government [Available from: https://www.gov.scot/policies/local-government/ accessed 5 Jan 2021.
41. The Scottish Government. Coronavirus (COVID-19): local protection levels 2021 [Available from: https://www.gov.scot/publications/coronavirus-covid-19-protection-levels/pages/overview/ accessed 5 Jan 2021.
42. The Scottish Government. Scottish Government Urban Rural Classification 2016. Edinburgh, 2018.
43. Stock SJ, McAllister D, Vasileiou E, et al. COVID-19 in Pregnancy in Scotland (COPS): protocol for an observational study using linked Scottish national data. *BMJ Open* 2020;10(11):e042813. doi: 10.1136/bmjopen-2020-042813 [published Online First: 2020/11/28]
44. Docherty AB, Harrison EM, Green CA, et al. Features of 20 133 UK patients in hospital with covid-19 using the ISARIC WHO Clinical Characterisation Protocol: prospective observational cohort study. *BMJ* 2020;369:m1985. doi: 10.1136/bmj.m1985 [published Online First: 2020/05/24]
45. Fine JP, Gray RJ. A Proportional Hazards Model for the Subdistribution of a Competing Risk. *Journal of the American Statistical Association* 1999;94(446):496-509. doi: 10.1080/01621459.1999.10474144
46. Naimi AI, Cole SR, Kennedy EH. An introduction to g methods. *Int J Epidemiol* 2017;46(2):756-62. doi: 10.1093/ije/dyw323 [published Online First: 2017/01/01]
47. Jackson JW, VanderWeele TJ. Intersectional decomposition analysis with differential exposure, effects, and construct. *Soc Sci Med* 2019;226:254-59. doi: 10.1016/j.socscimed.2019.01.033 [published Online First: 2019/02/17]
48. Knol MJ, VanderWeele TJ. Recommendations for presenting analyses of effect modification and interaction. *Int J Epidemiol* 2012;41(2):514-20. doi: 10.1093/ije/dyr218 [published Online First: 2012/01/19]
49. Lakha F, Gorman DR, Mateos P. Name analysis to classify populations by ethnicity in public health: validation of Onomap in Scotland. *Public Health* 2011;125(10):688-96. doi: 10.1016/j.puhe.2011.05.003 [published Online First: 2011/09/13]
50. Wilson N, Barnard LT, Summers JA, et al. Differential mortality rates by ethnicity in 3 influenza pandemics over a century, New Zealand. *Emerg Infect Dis* 2012;18(1):71-7. doi: 10.3201/eid1801.110035 [published Online First: 2012/01/20]

For peer review only

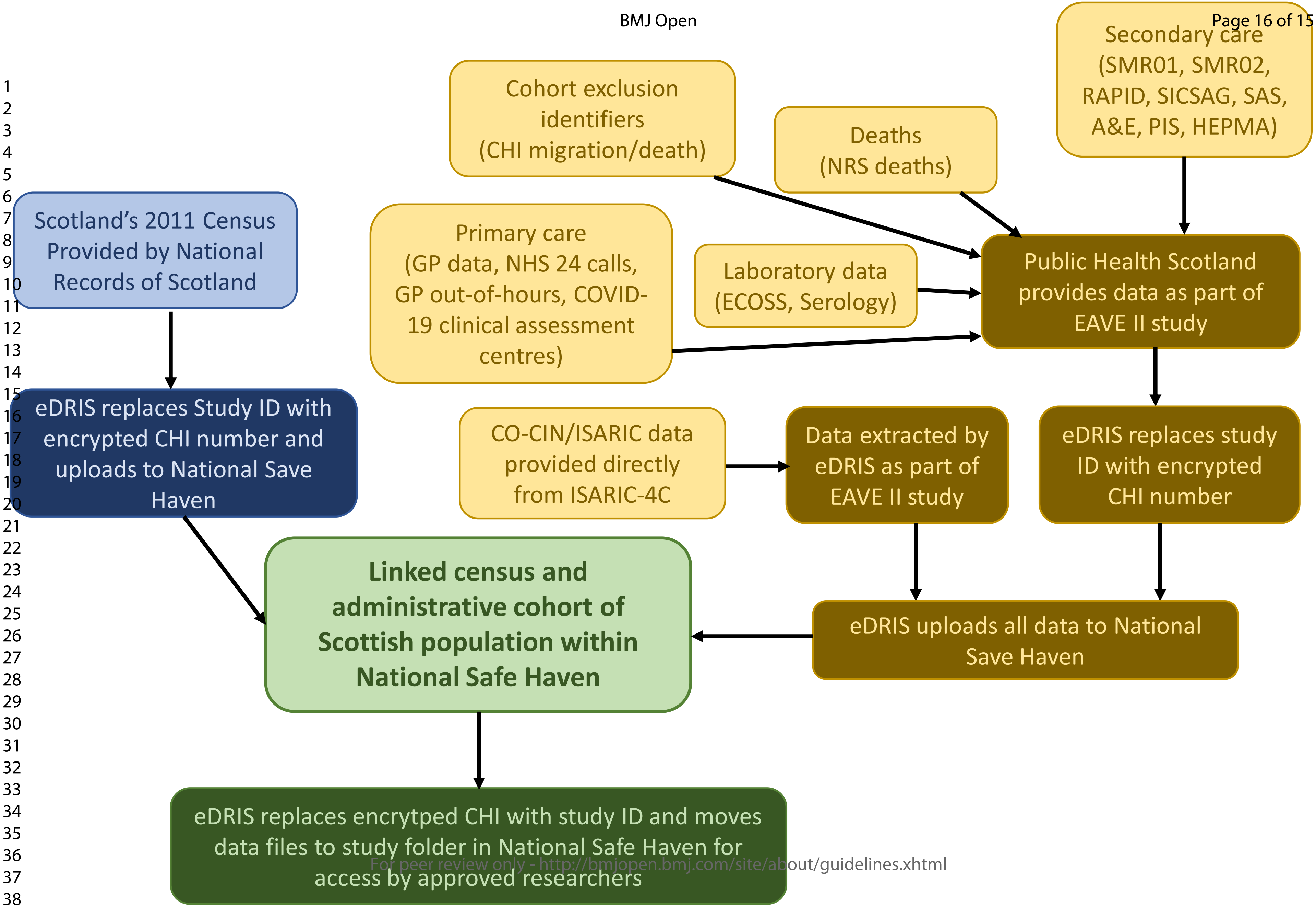

For peer review only - http://bmjopen.bmj.com/site/about/guidelines.xhtml

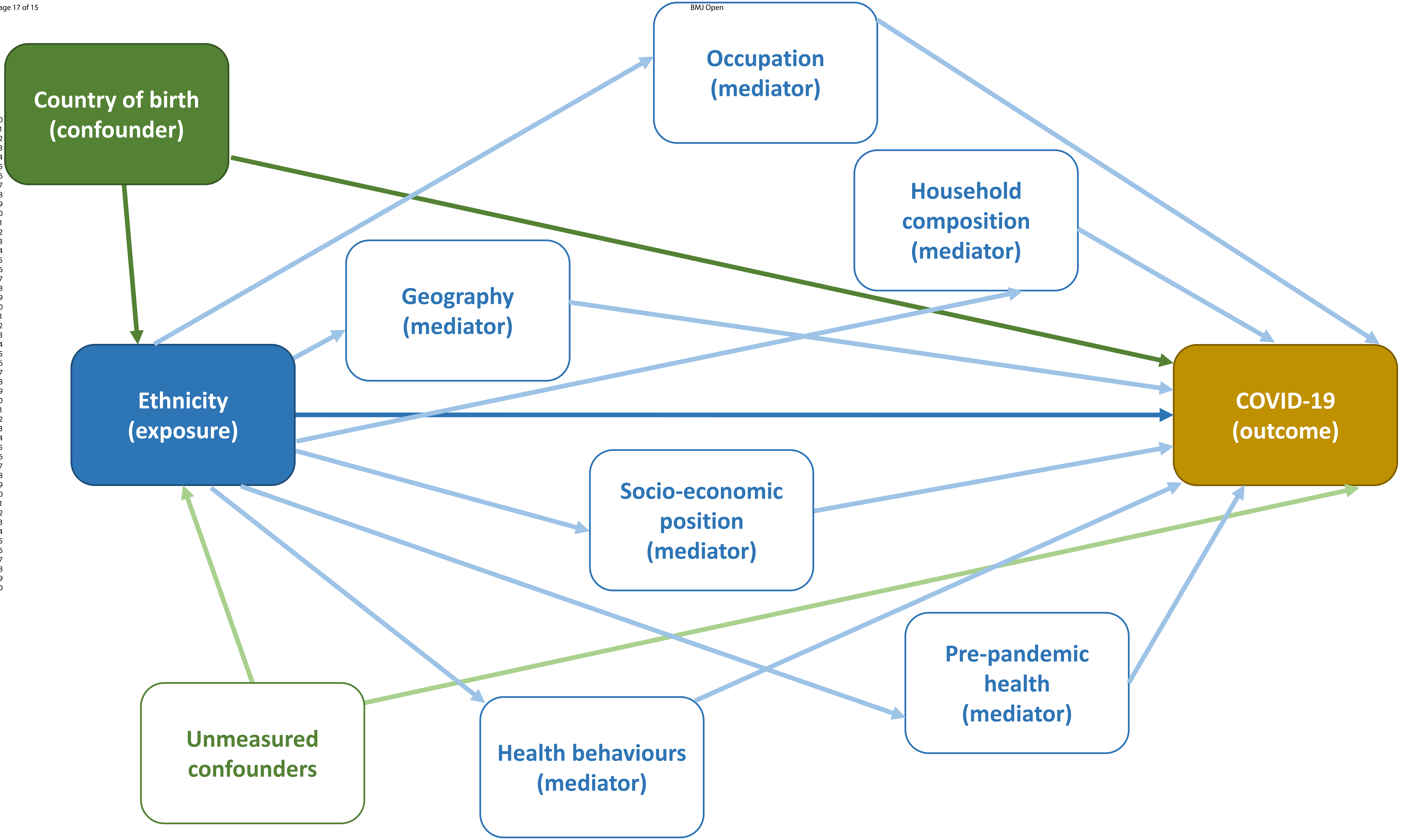
Country of birth (confounder)
Occupation (mediator)
Household composition (mediator)
Geography (mediator)
Ethnicity (exposure)
COVID-19 (outcome)
Socio-economic position (mediator)
Pre-pandemic health (mediator)
Unmeasured confounders
Health behaviours (mediator)

Ethnic and social inequalities in COVID-19 outcomes in Scotland: protocol for early pandemic evaluation and enhanced surveillance of COVID-19 (EAVE II)

Journal:	*BMJ Open*
Manuscript ID	bmjopen-2021-048852.R1
Article Type:	Protocol
Date Submitted by the Author:	30-Jun-2021
Complete List of Authors:	Henery, Paul; University of Glasgow, MRC/CSO Social and Public Health Sciences Unit; Public Health Scotland<br>Vasileiou, Eleftheria; The University of Edinburgh, Usher Institute<br>Hainey, Kirsten ; University of Glasgow, MRC/CSO Social & Public Health Sciences Unit<br>Buchanan, Duncan; Public Health Scotland<br>Harrison, Ewen ; University of Edinburgh<br>Leyland, Alastair; MRC/CSO Social and Public Health Sciences Unit, University of Glasgow<br>Alexis, Thomas; Scottish Government<br>Robertson, Chris; University of Strathclyde, Department of Mathematics and Statistics<br>Agrawal, Utkarsh; University of St Andrews, School of Medicine<br>Ritchie, Lewis; Aberdeen University, General Practice and Primary Care<br>Stock, Sarah; The University of Edinburgh Usher Institute of Population Health Sciences and Informatics, Centre for Medical Informatics; Public Health Scotland,<br>McCowan, Colin ; University of St. Andrews<br>Docherty, Annemarie; University of Edinburgh, Anaesthesia and Critical Care<br>Kerr, Steven; University of Edinburgh<br>Marple, James; University of Edinburgh<br>Wood, Rachael; NHS National Services Scotland, Information Services Division; University of Edinburgh<br>Moore, Emily; Public Health Scotland<br>Simpson, Colin; Victoria University of Wellington; University of Edinburgh<br>Sheikh, Aziz; University of Edinburgh, Division of Community Health Sciences<br>Katikireddi, Srinivasa; University of Glasgow
**Primary Subject Heading**:	Epidemiology
Secondary Subject Heading:	Public health, Sociology, Infectious diseases
Keywords:	COVID-19, EPIDEMIOLOGY, Protocols & guidelines < HEALTH SERVICES ADMINISTRATION & MANAGEMENT, Public health < INFECTIOUS DISEASES

For peer review only - http://bmjopen.bmj.com/site/about/guidelines.xhtml

For peer review only - http://bmjopen.bmj.com/site/about/guidelines.xhtml

I, the Submitting Author has the right to grant and does grant on behalf of all authors of the Work (as defined in the below author licence), an exclusive licence and/or a non-exclusive licence for contributions from authors who are: i) UK Crown employees; ii) where BMJ has agreed a CC-BY licence shall apply, and/or iii) in accordance with the terms applicable for US Federal Government officers or employees acting as part of their official duties; on a worldwide, perpetual, irrevocable, royalty-free basis to BMJ Publishing Group Ltd ("BMJ") its licensees and where the relevant Journal is co-owned by BMJ to the co-owners of the Journal, to publish the Work in this journal and any other BMJ products and to exploit all rights, as set out in our licence.

The Submitting Author accepts and understands that any supply made under these terms is made by BMJ to the Submitting Author unless you are acting as an employee on behalf of your employer or a postgraduate student of an affiliated institution which is paying any applicable article publishing charge ("APC") for Open Access articles. Where the Submitting Author wishes to make the Work available on an Open Access basis (and intends to pay the relevant APC), the terms of reuse of such Open Access shall be governed by a Creative Commons licence – details of these licences and which Creative Commons licence will apply to this Work are set out in our licence referred to above.

Other than as permitted in any relevant BMJ Author's Self Archiving Policies, I confirm this Work has not been accepted for publication elsewhere, is not being considered for publication elsewhere and does not duplicate material already published. I confirm all authors consent to publication of this Work and authorise the granting of this licence.

For peer review only

Ethnic and social inequalities in COVID-19 outcomes in Scotland: protocol for early pandemic evaluation and enhanced surveillance of COVID-19 (EAVE II)

Paul M. Henery [1, 2]
Eleftheria Vasileiou [3]
Kirsten J. Hainey [1]
Duncan Buchanan [2]
Ewen M. Harrison [3]
Alastair H. Leyland [1]
Thomas Alexis [4]
Chris Robertson [5]
Utkarsh Agrawal [6]
Lewis D. Ritchie [7]
Sarah J. Stock [3]
Colin McCowan [6]
Annemarie Docherty [3]
Steven Kerr [3]
James Marple [2]
Rachael Wood [2, 3]
Emily Moore [2]
Colin R. Simpson [8]
Aziz Sheikh [3]
S. Vittal Katikireddi [1, 2]

[1] MRC/CSO Social & Public Health Sciences Unit, University of Glasgow, UK
[2] Public Health Scotland, UK
[3] University of Edinburgh, UK
[4] Scottish Government, UK
[5] University of Strathclyde, UK
[6] University of St Andrews, UK
[7] University of Aberdeen, UK
[8] Victoria University of Wellington, New Zealand

Corresponding author:
Srinivasa Vittal Katikireddi
vittal.katikireddi@glasgow.ac.uk

MRC/CSO Social and Public Health Sciences Unit
University of Glasgow
Berkeley Square
99 Berkeley Street
Glasgow
G3 7HR

Word count: 3,834

For peer review only

ABSTRACT

Introduction: Evidence from previous pandemics, and the current COVID-19 pandemic, has found that risk of infection/severity of disease is disproportionately higher for ethnic minority groups, and those in lower socio-economic positions. It is imperative that interventions to prevent the spread of COVID-19 are targeted towards high-risk populations. We will investigate the associations between social characteristics (such as ethnicity, occupation and socio-economic position) and COVID-19 outcomes and the extent to which characteristics/risk factors might explain observed relationships in Scotland.
The primary objective of this study is to describe the epidemiology of COVID-19 by social factors. Secondary objectives are to a) examine receipt of treatment and prevention of COVID-19 by social factors; b) quantify ethnic/social differences in adverse COVID-19 outcomes; c) explore potential mediators of relationships between social factors and SARS-CoV-2 infection/COVID-19 prognosis; d) examine whether occupational COVID-19 differences differ by other social factors, and e) assess quality of ethnicity coding within NHS datasets.

Methods and analysis: We will use a national cohort comprising the adult population of Scotland who completed the 2011 Census and were living in Scotland on March 31st 2020 (~4.3 million people). Census data will be linked to the EAVE II cohort consisting of primary/secondary care, laboratory data and death records. Sensitivity/specificity and positive/negative predictive values will be used to assess coding quality of ethnicity. Descriptive statistics will be used to examine differences in treatment and prevention of COVID-19. Poisson/Cox regression analyses, and mediation techniques will examine ethnic and social differences, and drivers of inequalities in COVID-19. Effect modification (on additive and multiplicative scales) between key variables (such as ethnicity and occupation) will be assessed.

Ethics and dissemination: Ethical approval was obtained from the National Research Ethics Committee, South East Scotland 02. We will present findings of this study at international conferences, in peer-reviewed journals and to policymakers.

STRENGTHS AND LIMITATIONS OF THIS STUDY

- This study uses a near-complete cohort of the Scottish population, providing considerable statistical power to investigate social inequalities in COVID-19.
- Linkage of the Scottish Census to administrative data allows for evaluation of differences in COVID-19 across specific occupational classes, which is not available in any other dataset in Scotland.
- Given the Census data are from 2011 and are being linked to data from 2020-21, some occupational and social data may be out-of-date.
- Individuals who moved to Scotland post-Census will not be included in all analyses, for the same reasons as above.

For peer review only

INTRODUCTION

Historically, pandemics have disproportionately affected specific groups within the population. For example, during the Spanish influenza pandemic of 1918, individuals from poorer socio-economic positions experienced higher incidence of influenza in the United States.[1] These findings have been replicated in various international settings using data from the 1918 pandemic.[2] Similar patterns were observed following the H1N1 influenza pandemic in 2009 in Canada, with an increased risk of hospitalisation amongst individuals with lower educational attainment or resident in areas of greater deprivation.[3] English data demonstrated a socio-economic gradient in mortality during the 2009 pandemic, with the highest risk of death observed in those living in deprived areas.[4 5] Ethnicity was also identified as a risk factor for mortality during the 2009 pandemic, with those in non-White ethnic groups at increased risk of mortality from H1N1 influenza compared to White populations in the UK.[5] The mechanisms underlying these socio-economic patterns have been hypothesised as resulting from a combination of different exposure and susceptibility to infection, and differing access to healthcare services.[6] Understanding the role of ethnic, socio-economic and occupational risk factors in both risk and outcome of infection during a pandemic is essential for health service planning, targeting preventative measures and informing future modelling efforts.

The Severe Acute Respiratory Syndrome coronavirus-2 (SARS-CoV-2) and its resulting disease (COVID-19) is spreading rapidly worldwide, causing a high burden of mortality and morbidity.[7] The differential risks of infection in ethnic minority groups have been examined during the COVID-19 pandemic. Epidemiological analyses within England have found higher risks among some minority ethnic groups, both for risk of infection and for the consequences of infection.[8-12] These findings have been supported by international evidence, which suggests individuals from Black and South Asian ethnic groups are at increased risk of experiencing COVID-19 and its resultant harms.[11 13]

Emerging evidence suggests that marked socio-economic inequalities in risk and outcome of COVID-19 exist, yet empirical evidence describing the role of socio-economic factors in Scotland is limited.[10] Initial analyses in Scotland have been subject to considerable misclassification of ethnicity (due to systematically incomplete data within health records) and limited by using a broad three-category classification of ethnicity in a small case-control sample;[14] however, survey-based research with more specific ethnicity classifications has suggested that ethnic minorities may be more hesitant to be vaccinated against COVID-19.[15] Analyses have therefore shown inconsistent findings. Understanding how sociodemographic factors are related to infection risk and prognosis, as well as monitoring inequalities by area-level (such as indices of multiple deprivations) and individual/household-level (such as social class or household tenure) socio-economic position, is essential for health service planning, targeting prevention efforts and informing future modelling efforts. In addition, our understanding of why inequalities in COVID-19 outcomes exist remain limited.[16] For example, differences in occupation, household composition and healthcare could operate as important mediating mechanisms, which could potentially be targeted to mitigate ethnic inequalities in COVID-19.

The Early Assessment of Vaccine and Anti-Viral Effectiveness II (EAVE II) study[17-19] is an ongoing project which tracks the epidemiology of COVID-19 in Scotland in real-time, and aims to evaluate the effectiveness of future vaccinations in reducing spread of the infection. Whilst the main EAVE II project will stratify COVID-19 rates and vaccine effectiveness by demographic characteristics such as age and region, it will not examine the role of ethnicity and social factors in detail. Linkage of the EAVE II dataset to other administrative sets, in particular the 2011 Scotland Census, allows the assessment of the role of social and ethnic inequalities on risk of SARS-CoV-2 infection and subsequent disease outcomes. This provides better understanding of groups at higher risk from COVID-19 within the Scottish population compared to administrative data sources (which typically

For peer review only

lack detailed information about social characteristics), yielding valuable information for clinicians and healthcare planners as the pandemic continues to evolve. Learning from this pandemic may also yield longer term lessons for public health policy.

Aim and objectives

This EAVE II sub-study aims to quantify social inequalities in COVID-19 related outcomes in Scotland using linked Census and administrative data, as well as examining the mechanisms through which inequalities in COVID-19 arise.

Our primary objective is to describe the epidemiology of COVID-19 by: i) ethnicity; ii) socio-economic position; iii) occupation; and iv) residential circumstances.

In addition, we have five secondary objectives:

a. To monitor receipt of COVID-related treatment and preventive interventions across social and ethnic groups
b. To quantify ethnic and social differences in the risk of adverse outcomes (hospital admission, critical care admission and mortality) from COVID-19
c. To explore the potential contribution of pre-existing health conditions and risk factors to ethnic and social inequalities in COVID-19 and prognosis
d. To investigate if occupational (and potentially socio-economic or ethnic) differences in COVID-19 risks are modified by other social factors.
e. To assess the quality of ethnicity coding within NHS administrative datasets

Secondary objective b will be examined for three groups defined by experiencing: (i) laboratory confirmed SARS-CoV-2 infection; (ii) clinically diagnosed SARS-CoV-2 infection; (iii) serological evidence of previous infection with SARS-CoV-2.

METHODS AND ANALYSIS

Study design and population

We will perform analyses on a national prospective cohort of the Scottish population, including all individuals who meet the following criteria: a) alive on the 1st March 2020; b) resident in Scotland on that date; and c) present in the 2011 Census in Scotland. A start date of 1st March 2020 has been selected as the first case of laboratory confirmed SARS-CoV-2 in Scotland was identified on 2nd March 2020.[20] Analyses will focus on the adult population aged 16 years and over, with some analyses limited to older age groups (e.g. aged 25+ years on the Census 2011 date when analysing highest education level). We will also conduct analyses that include individuals not present in the 2011 Census when they are of specific policy interest – for example, to look at vaccination uptake.

Databases

The Community Health Index (CHI) is a unique numerical identifier, assigned to every individual registered to receive healthcare in Scotland, which can be used to identify and link all healthcare interactions and records for an individual.[21] Linkage of these administrative datasets with the 2011 Census data will allow the examination of clinically diagnosed and laboratory confirmed SARS-CoV-2 infection, deaths and potential confounders (Fig. 1). The CHI is mandatory for anyone receiving medical treatment.[21] Furthermore, individuals known to have emigrated (through GP de-

For peer review only

registration) prior to the pandemic will be excluded. Data linkage and analyses will take place within the National Safe Haven, a secure and trusted research environment (TRE).[22]

Census

In Scotland, the national Census is conducted every 10 years by means of a mandatory survey sent to every household in the country.[23] The most recent Census was conducted in 2011 and forms the population spine of our cohort. The Census extract for this study contains demographic information including ethnic group, religion and country of birth, marital status, socio-economic position (SEP) indicators such as highest educational attainment, employment status and occupation, household information including persons per room, tenure and number of cars, and variables on pre-pandemic health status identifying long-term illnesses and disability status. Census data are necessary to identify our exposures of interest, as well as to study potential mediating pathways and effect modification. The necessary information required from the Census (e.g. ethnicity, occupation) is not reliably available from other sources. While ethnicity is recorded in some health datasets in Scotland, it is often missing, reported inconsistently across datasets, and not always based on self-report, which is considered the gold standard for establishing ethnicity.[24]

Laboratory data

To identify individuals with confirmed SARS-CoV-2 infection, positive viral reverse transcriptase polymerase chain reaction (RT-PCR) results will be confirmed using Electronic Communication of Surveillance in Scotland (ECOSS) data, a national repository of diagnostic and reference laboratory data on testing for viral infections including COVID-19.[25] To identify individuals with serologically confirmed infection, serology data will be provided via the Enhanced Surveillance of COVID-19 in Scotland (ESoCiS) programme undertaken by Public Health Scotland (PHS) on behalf of the Scottish Government.[26]

Secondary care

The Scottish Morbidity Record (SMR), maintained by PHS, is the primary repository for data describing hospital admissions in Scotland.[27] We will use data from SMR01, which details general inpatient admissions, and SMR02, which details obstetric admissions. We will include data from the Rapid Preliminary Inpatient Data (RAPID) dataset. The RAPID data are collected quickly, in an unprocessed state and are therefore available more quickly than SMR data,[28] which is necessary given the pertinent nature of our study. Data about intensive care unit admissions in adults will be included by linking data from the Scottish Intensive Care Society Audit Group (SICSAG).[29] These datasets use International Classification of Diseases (ICD-10) codes to record an individual's diagnoses. ICD-10 codes will be used to identify a clinical diagnosis of COVID-19 in the absence of a positive test result for those admitted to hospital with diagnosed COVID-19. Scottish Ambulance Service (SAS) and Accident and Emergency (A&E) records, also provided by PHS, will be used to identify people with clinically suspected COVID-19.

The Prescribing Information System (PIS) is the repository for all prescriptions requested for individuals by general practices,[30] and the Hospital Electronic Prescribing and Medicines Administration (HEPMA) is the equivalent for prescribing and administration of medicine in a hospital setting.[31] Both of these datasets use the British National Formulary (BNF), a standardised medication classification system.[32] We will use BNF codes to identify prescribed drugs or administered medicine used to treat COVID-19, identify comorbidities (such as diabetes and hypertension) and allow the study of longer-term consequences of COVID-19.

For peer review only - http://bmjopen.bmj.com/site/about/guidelines.xhtml

Primary care

Just under 100% of the population of Scotland is registered with a general practice (GP), which provides free-at-point-of-delivery primary healthcare services.[33] NHS 24 is the Scottish national telephone service for the general public, which provides clinical triage and advice for those experiencing non-critical ill health during the out-of-hours period. This was expanded to provide advice to individuals with suspected COVID-19 at any time during the pandemic.[34] We will use GP consultation data and NHS24 out-of-hours data to identify clinically suspected COVID-19. In addition, data from the recently established COVID-19 clinical assessment centres will also be included for this purpose.

Deaths

National Records for Scotland (NRS) provides data about deaths attributed to COVID-19, which will be included in our dataset.[35] ICD-10 codes will be used to identify deaths occurring in individuals with clinically confirmed COVID-19 within 28 days of death (U07.1, U07.2).

Exposures and covariates

We will examine four categories of social factors in relation to the study's primary outcomes: ethnicity, socio-economic position (SEP), occupation and residential circumstances. Ethnicity will be categorised based on standard Census classifications,[36] self-reported in sixteen groups. We will try to analyse ethnicity using as detailed categorisations as possible, subject to having adequate numbers for meaningful statistical analysis. SEP will be classified at both area-level (the Scottish Index of Multiple Deprivation [SIMD]) and individual level (e.g. education level and housing tenure). The SIMD is a measure of the relative deprivation of broadly heterogeneous population-level "data zones" within Scotland, classifying deprivation using the following weighted domains: employment, income, health, crime, housing, education and access to services.[37] The SIMD is typically operationalised using either quintiles or deciles, with quintile/decile 1 referring to the most deprived 20% or 10% of the population, respectively. Marital status will consist of five categories as per the Census: single (never married), married, separated, divorced and widowed.[38] Housing tenure will consist of seven categories as per the Census: owned (outright), owned (mortgage), social rented (council), social rented (other), private rented (landlord/letting agency), private rented (other/rent-free), or communal.[39] Occupation will be measured using the Standard Occupational Classification 2000, consisting of major, sub-major, minor and unit group levels.[40] Employment status, weekly hours worked, and type of industry (coded using Standardised Industrial Classification, SIC) will be used to explore potential differential workplace exposure. Residential circumstances will be classified using the following parameters: housing tenure, persons per room, household size and age range of household (difference between oldest and youngest permanent occupant).

The following additional characteristics will be used to address confounding, explore mediation and for assessing effect modification. Cultural factors will be investigated, including religion, and English language ability via a four-point Likert scale (very well, well, not well, not at all). Geographical variables will include 32 local authorities (LA) comprising local government in Scotland. Whilst these LA have some devolved powers, primarily in service provision,[41] the most pertinent local factor for this study is that COVID-19 mitigation levels are, at the time of writing, allocated by a LA-level tiering system.[42] We will explore the role of rurality using the Scottish Government Urban Rural Classification (URC), which can be used as a simple two-fold measure (defining "rurality" as areas with a population of <3,000) at its simplest, or an eight-fold measure at its most complex).[43] Pre-pandemic health status will be assessed on the basis of general self-reported health, captured using a five-point Likert scale (very good, good, fair, bad, very bad), and the presence of the following

For peer review only

additional self-reported health circumstances as recorded in the Census: long-term condition, mental health condition, other condition, hearing impairment, visually impairment, learning disability, developmental disorder, and physical disability. Behavioural risk factors (such as smoking status and body mass index) and health conditions (including pregnancy) will also be identified from GP and hospital data.

Outcome definitions

The primary outcomes of this study will be laboratory confirmed SARS-CoV-2 infection, clinically diagnosed COVID-19, and serologically confirmed evidence of previous SARS-CoV-2 infection. Laboratory confirmed SARS-CoV-2 infection will be defined as positive RT-PCR via the ECOSS dataset. Serologically confirmed SARS-CoV-2 infection will be defined as positive antibodies on serology testing as identified through ESoCiS data, reflecting immunological evidence of previous infection. Clinically diagnosed COVID-19 disease will be defined as the requisite ICD-10 code being recorded on discharge from hospital via SMR, intensive care via SICSAG, or on NRS death records. Clinically suspected COVID-19 disease will be used as a secondary outcome in the absence of laboratory confirmed COVID-19 and recorded via SAS or A&E attendance records, GP consultation or out-of-hours records, COVID-19 clinical assessment centre data, or NHS 24 logs. Given testing practices are changing over time, we will conduct sensitivity analyses by stratifying analyses by time period and consider weighting analyses to account for differential testing practices.

Secondary outcomes include uptake of vaccination for COVID-19, as recorded in GP records or the vaccine delivery app, and being prescribed treatment for COVID-19 either via the PIS or HEPMA. We will also examine adverse outcomes following COVID-19 infection: admission to hospital, critical care admission, or death. Hospital admission or death will be defined on the basis of either COVID-19 (laboratory confirmed or clinically diagnosed) recorded on a hospital admission in SMR01 or death record (using ICD10 codes U07.1, U07.2,) or positive test, or admission in RAPID within 14 days of a SARS-CoV-2 RT-PCR positive test.[44] Critical care admissions will be defined on the basis of presence on the SICSAG database and a relevant hospital admission (using ICD10 codes U07.1, U07.2), or positive test.

Statistical analysis

Epidemiology of COVID-19 by ethnicity and social factors

We will describe the epidemiology of COVID-19 by ethnic and social factors, specifically in relation to: a) incidence of confirmed SARS-CoV-2 infection; b) incidence of clinically diagnosed disease; and c) evidence of previous infection. We will use summary statistics to describe the cohort by presence and absence of confirmed, diagnosed and suspected COVID-19. We will perform a descriptive analysis of the number of cases by exposure groups in the first instance, to determine the level of disaggregation feasible. In addition to analysing inequalities by ethnicity and social factors, we will also investigate the inequalities by country of birth (i.e. migration-related inequalities).
Poisson regression with robust standard errors will be used to estimate risk ratios for the risk of infection across exposure groups. We will consider age and sex to be key potential confounders for all analyses, with adjustment for local authority also planned for most analyses (to account for the differential spread of the infection across geographies, and local mitigation levels). If case numbers allow, we will examine the incidence of confirmed and suspected COVID-19 by sex (and for ethnicity, country of birth). We can assess whether findings are robust to more stringent definitions of confirmed and suspected infection, e.g. by restricting the definition of a confirmed case to one with positive viral RT-PCR, and restricting the definition of a suspected case to exclude people with a negative viral RT-PCR result.

For peer review only

Surveillance of preventative treatment and interventions for COVID-19 across social and ethnic factors

We will describe treatment for and prevention of COVID-19 by ethnicity and other social factors (e.g. socio-economic position, occupation), especially in relation to vaccination uptake. We will tabulate those who have been vaccinated as a proportion of the total eligible in different social subgroups, from when the vaccine is available and delivered nationally. This will be monitored over time to investigate whether there is a change in vaccination rates for different subgroups. If we have sufficient numbers of people vaccinated, we will also stratify these analyses by demographic factors. Similarly, we will also investigate the potential ethnic and other social differences in treatment for COVID-19 during acute infection and post-COVID via GP prescribing or hospital-administered medical care.

Inequalities in risk of adverse health outcomes from COVID-19

We will quantify ethnic and social inequalities in the risk of adverse outcomes (hospital admission, critical care admission and mortality) from COVID-19. We will perform a descriptive analysis of the number of cases by exposure group in the first instance, to help determine the level of disaggregation feasible. We will follow a previously published approach[45] to analyse COVID-19 survival outcomes using Cox proportional hazards models from the date of a positive test result (or first date of diagnosis). We will conduct sensitivity analyses excluding people resident in care homes at the study start date. We will consider Fine & Gray competing risks models (to account for non-COVID-19 deaths) for sensitivity analysis[46] or alternatively by applying inverse probability weights to address competing risks.

Mediation analyses to explore drivers of inequalities in COVID-19

We will explore the contribution of several potential mediators of ethnic and social inequalities in COVID-19. The extent of these analyses will be determined by the magnitude of any observed inequalities in COVID-19 outcomes. Potential mediators to be investigated for ethnic inequalities include occupation, socio-economic position, cultural factors (such as language ability and country of birth) and pre-pandemic health (assessed through census, GP records and hospitalisations data). We will conduct cross-tabulations of the relationships between exposures, potential mediators and outcomes, followed by a set of nested regression models for the same purpose. Following this, we will pursue more formal mediation analyses. We expect to use G methods,[47] such as marginal structural modelling, to estimate the direct and indirect effect of ethnicity mediated through the mediating factors. We will investigate the extent to which inequalities might arise from differential exposure compared to differential susceptibility.[48] Our analysis will be informed by a Directed Acyclic Graph (DAG) (Fig. 2). Given we are uncertain about whether the country of birth and geography are confounders or mediators, we will conduct sensitivity analyses without adjusting for these variables. In addition to analysing inequalities by ethnicity, we will investigate inequalities by country of birth to explore migration-related inequalities. We will consider conducting similar mediation analyses for socio-economic inequalities in COVID-19, depending on the extent of inequalities observed.

Effect modification

We will investigate whether interactions exist for key combinations of exposure variables, such as ethnicity, socio-economic position and occupation. We will study whether occupational risks of COVID-19 differ by ethnic group. Occupation will be assessed on the basis of Standard Occupational Classification 2000 codes, aggregated from three-digit codes to a level that meets disclosure control

For peer review only

requirements. We will perform descriptive analysis of the number of cases by occupation initially, followed by cross-tabulation by ethnicity. We will then estimate relative risks for outcomes of interest and check for evidence of effect modification on an additive and multiplicative scale. Should we observe evidence that occupational risk differs by ethnicity, we will calculate relative excess risks due to interaction.[49] Similar analyses will be conducted to assess potential effect modification for key combinations of exposure groups, as prioritised by our policy partners.

Assessment of quality of ethnicity coding

We will assess the quality of ethnicity coding within NHS administrative datasets, in order to assess whether, in the absence of Census data, administrative data can be reliably used to study ethnic inequalities. We will start by assessing the proportion of information on ethnicity missing within each dataset. Following this, we will consider the 2011 Census to be our 'gold standard' dataset for comparison purposes. We will quantify the prevalence of different ethnic groups within each dataset and assess the sensitivity, specificity, positive predictive value and negative predictive value, following the methods used in previous validation studies.[50] If numbers of cases allow, we will stratify analyses by broad age group, sex and country of birth.

Sample size

Accounting for non-linkage and non-participation in the Census (via migration or birth after the Census or otherwise), we estimate that the size of the cohort will be 4.3 million people. We are confident based on research from previous pandemics in similar-sized populations[51] that there will be sufficient statistical power to perform our planned analyses. For example, for the African ethnic group which is one of the least frequent ethnic groups in Scotland, considering COVID-19 deaths (the least common outcome), we anticipate there being approximately 30 COVID-19 deaths from the first wave in the African ethnic group which exceeds the number of events in previous completed analyses. Utlimately, the total number of events is dependent on the trajectory of the COVID-19 pandemic in Scotland, and statistical power will be assessed on an outcome-by-outcome basis.

Patient and public involvement (PPI)

An EAVE II PPI group has been established, which includes diverse ethnic and socio-economic representation. Study plans have been discussed with the group and this has confirmed the importance of investigating a broad range of social factors (including socio-economic position, as well as ethnicity). The study design was initially developed by the study investigators without PPI due to delays in being able to establish the PPI group, but more substantive input into the creation of detailed analysis plans is now ongoing. Findings will be discussed regularly with the group and revised on the basis of their input accordingly. The PPI group will also inform the development of dissemination plans.

ETHICS AND DISSEMINATION

This study, as part of the wider EAVE II project, has been approved by the National Research Ethics Committee, South East Scotland 02. The study has also received approval from the Public Benefit and Privacy Panel for Health and Social Care (2021-0015), and approval from the Statistics Public Benefit and Privacy Panel (2021-0115). Findings from this study will be published in peer-reviewed journals and presented at international conferences. Analyses will be presented to policymakers within UK and Scottish governments and to relevant public health practitioners in Scotland throughout the course of the study. STROBE and RECORD guidelines will be used in reporting the findings of the study.

For peer review only

AUTHORS' CONTRIBUTIONS

SVK designed the study. PMH, EV, DB, KJH and CRS critically revised the study design. PMH drafted the manuscript. EV, KJH, DB, EMH, AHL, TA, CR, UA, LDR, SJS, CM, AD, SK, JM, RW, EM, AS and SVK critically revised the manuscript. SVK obtained funding for the study.

FUNDING STATEMENT

This study is being funded by the UKRI Centre on the Dynamics of Ethnicity 4 (ES/W000849/1). PMH, SVK, AHL and KH are funded by the Medical Research Council (MC_UU_00022/2) and Scottish Government Chief Scientist Office (SPHSU17). SVK is funded by an NRS Senior Clinical Fellowship (SCAF/15/02). EAVE II is funded by the Medical Research Council (MR/R008345/1) with the support of BREATHE - The Health Data Research Hub for Respiratory Health [MC_PC_19004], which is funded through the UK Research and Innovation Industrial Strategy Challenge Fund and delivered through Health Data Research UK; with additional support from the Scottish Government DG Health and Social Care.

COMPETING INTERESTS STATEMENT

SVK is co-chair of the Scottish Government's Expert Reference Group on ethnicity and COVID-19 and a member of the UK Government's Scientific Advisory Group on Emergencies (SAGE) subgroup on ethnicity and COVID-19. AS is a member of the Scottish Government's Chief Medical Officer's COVID-19 Advisory Group, the Scottish Government's Expert Reference Group on ethnicity and COVID-19 and the UK Government's Subgroup on Risk Stratification of the New and Emerging Respiratory Virus Threats Group (NERVTAG). LR acts as a medical advisor to Scottish Government on primary care and public health, including vaccination issues. CRS reports grants from the UK National Institute for Health Research, Medical Research Council and New Zealand Health Research Council, and The Ministry of Business, Innovation and Employment.

FIGURE LEGENDS

Fig. 1: Data flow diagram for linkage of census and administrative data comprising cohort
Blue box: Census data
Yellow box: Administrative data
Green box: Linked data
Dark background: Data processing

Fig. 2: Directed Acyclic Graph (DAG) of the causal pathways between ethnicity and COVID-19
Yellow box: Outcome
Blue box: Exposure
Green box: Confounder
White box with green outline: Confounder (unmeasured)
White box with blue outline: Mediator
Blue arrow: Main effect
Green arrow: Confounding
Light green arrow: Unmeasured confounding
Light blue arrow: Mediated effect

REFERENCES

For peer review only

1. Sydenstricker E. The incidence of influenza among persons of different economic status during the epidemic of 1918. *Public Health Reports (1896-1970)* 1931;46(4):154-70.
2. Bambra C, Riordan R, Ford J, et al. The COVID-19 pandemic and health inequalities. *J Epidemiol Community Health* 2020;74(11):964-68. doi: 10.1136/jech-2020-214401 [published Online First: 2020/06/15]
3. Lowcock EC, Rosella LC, Foisy J, et al. The social determinants of health and pandemic H1N1 2009 influenza severity. *Am J Public Health* 2012;102(8):e51-8. doi: 10.2105/AJPH.2012.300814 [published Online First: 2012/06/16]
4. Rutter PD, Mytton OT, Mak M, et al. Socio-economic disparities in mortality due to pandemic influenza in England. *Int J Public Health* 2012;57(4):745-50. doi: 10.1007/s00038-012-0337-1 [published Online First: 2012/02/03]
5. Zhao H, Harris RJ, Ellis J, et al. Ethnicity, deprivation and mortality due to 2009 pandemic influenza A(H1N1) in England during the 2009/2010 pandemic and the first post-pandemic season. *Epidemiol Infect* 2015;143(16):3375-83. doi: 10.1017/S0950268815000576 [published Online First: 2015/04/09]
6. P. B, A. R, S. E, et al. Pandemic influenza planning in the United States from a health disparities perspective. *Emerg Infect Dis* 2008;14(5):709-15.
7. World Health Organization. Coronavirus disease 2019 (COVID-19): situation report 91, 2020.
8. Aldridge RW, Lewer D, Katikireddi SV, et al. Black, Asian and Minority Ethnic groups in England are at increased risk of death from COVID-19: indirect standardisation of NHS mortality data. *Wellcome Open Res* 2020;5:88. doi: 10.12688/wellcomeopenres.15922.2 [published Online First: 2020/07/10]
9. Intensive Care National Audit & Research Centre. ICNARC report on COVID-19 in critical care: England, Wales and Northern Ireland. London, 2020.
10. Niedzwiedz CL, O'Donnell CA, Jani BD, et al. Ethnic and socioeconomic differences in SARS-CoV-2 infection: prospective cohort study using UK Biobank. *BMC Med* 2020;18(1):160. doi: 10.1186/s12916-020-01640-8 [published Online First: 2020/05/30]
11. White C, Nafilyan V. Coronavirus (COVID-19) related deaths by ethnic group, England and Wales: 2 March 2020 to 10 April 2020. In: Statistics OfN, ed., 2020.
12. Williamson E, Walker AJ, Bhaskaran K, et al. OpenSAFELY: factors associated with COVID-19-related hospital death in the linked electronic health records of 17 million adult NHS patients. *Preprint* 2020 doi: 10.1101/2020.05.06.20092999
13. Sze S, Pan D, Nevill CR, et al. Ethnicity and clinical outcomes in COVID-19: A systematic review and meta-analysis. *EClinicalMedicine* 2020;29:100630. doi: 10.1016/j.eclinm.2020.100630 [published Online First: 2020/11/18]
14. McKeigue PM, Weir A, Bishop J, et al. Rapid Epidemiological Analysis of Comorbidities and Treatments as risk factors for COVID-19 in Scotland (REACT-SCOT): A population-based case-control study. *PLoS Med* 2020;17(10):e1003374. doi: 10.1371/journal.pmed.1003374 [published Online First: 2020/10/21]
15. Robertson E, Reeve KS, Niedzwiedz CL, et al. Predictors of COVID-19 vaccine hesitancy in the UK Household Longitudinal Study. *Preprint* 2021 doi: 10.1101/2020.12.27.20248899
16. Katikireddi SV, Lal S, Carrol ED, et al. Unequal impact of the COVID-19 crisis on minority ethnic groups: a framework for understanding and addressing inequalities. *J Epidemiol Community Health* 2021 doi: 10.1136/jech-2020-216061 [published Online First: 2021/04/23]
17. Simpson CR, Beever D, Challen K, et al. The UK's pandemic influenza research portfolio: a model for future research on emerging infections. *The Lancet Infectious Diseases* 2019;19(8):e295-e300. doi: 10.1016/s1473-3099(18)30786-2
18. Simpson CR, Robertson C, Vasileiou E, et al. Early Pandemic Evaluation and Enhanced Surveillance of COVID-19 (EAVE II): protocol for an observational study using linked Scottish national data. *BMJ Open* 2020;10(6):e039097. doi: 10.1136/bmjopen-2020-039097 [published Online First: 2020/06/23]

For peer review only

19. Simpson CR, Thomas BD, Challen K, et al. The UK hibernated pandemic influenza research portfolio: triggered for COVID-19. *The Lancet Infectious Diseases* 2020;20(7) doi: 10.1016/s1473-3099(20)30194-8
20. Filipe AdS, Shepherd J, Williams T, et al. Genomic epidemiology of SARS-CoV-2 spread in Scotland highlights the role of European travel in COVID-19 emergence. *Preprint* 2020 doi: 10.1101/2020.06.08.20124834
21. ISD Scotland. ISD Scotland data dictionary: CHI number [Available from: https://www.ndc.scot.nhs.uk/Dictionary-A-Z/Definitions/index.asp?Search=C&ID=128&Title=CHI%20Number accessed 5 Jan 2021.
22. NHS Scotland. Data Safe Haven 2021 [Available from: https://www.nhsresearchscotland.org.uk/research-in-scotland/data/safe-havens accessed 5 Jan 2021.
23. National Records of Scotland. What is Scotland's Census? 2018 [Available from: https://www.scotlandscensus.gov.uk/what-census accessed 5 Jan 2021.
24. NHS National Services Scotland. Measuring use of health services by equality group. NSS Information and Intelligence. Edinburgh, 2017.
25. Health Protection Scotland. Data and surveillance 2020 [Available from: https://www.hps.scot.nhs.uk/data/ accessed 5 Jan 2021.
26. Health Protection Scotland. Enhanced surveillance of coronavirus (COVID-19) 2020 [Available from: https://www.hps.scot.nhs.uk/a-to-z-of-topics/enhanced-surveillance-of-coronavirus-covid-19/ accessed 5 Jan 2021.
27. ISD Scotland. SMR datasets: episode management: SMR record type [Available from: https://www.ndc.scot.nhs.uk/Data-Dictionary/SMR-Datasets//Episode-Management/SMR-Record-Type/ accessed 5 Jan 2021.
28. Public Health Scotland. System Watch 2020 [Available from: https://www.isdscotland.org/Products-and-Services/System-Watch/ accessed 5 Jan 2021.
29. Scottish Intensive Care Society Audit Group. About SICSAG [Available from: https://www.sicsag.scot.nhs.uk/about/main.html accessed 5 Jan 2021.
30. ISD Scotland. National data catalogue: national datasets [Available from: https://www.ndc.scot.nhs.uk/National-Datasets/data.asp?SubID=9 accessed 5 Jan 2021.
31. eHealth. HEPMA 2015 [Available from: https://www.ehealth.scot/case-studies/hepma/ accessed 5 Jan 2021.
32. BNF Publications. Our organisation 2021 [Available from: https://www.bnf.org/about/ accessed 5th Jan 2021.
33. A. B, R. T, J. M. NHS number and the systems used to manage them: an overview for research users. In: CLOSER, ed. Resource Report. London, 2018.
34. NHS 24. When to phone 111 2019 [Available from: https://www.nhs24.scot/111/when-to-phone-111/ accessed 5 Jan 2021.
35. National Records of Scotland. Deaths involving coronavirus (COVID-19) in Scotland 2021 [Available from: https://www.nrscotland.gov.uk/statistics-and-data/statistics/statistics-by-theme/vital-events/general-publications/weekly-and-monthly-data-on-births-and-deaths/deaths-involving-coronavirus-covid-19-in-scotland accessed 5 Jan 2021.
36. National Records of Scotland. Ethnic group 2018 [Available from: https://www.scotlandscensus.gov.uk/variables-classification/ethnic-group accessed 5 Jan 2021.
37. The Scottish Government. Scottish Index of Multiple Deprivation 2020 2020 [Available from: https://www.gov.scot/collections/scottish-index-of-multiple-deprivation-2020/ accessed 5 Jan 2021.
38. National Records of Scotland. Marital status 2018 [Available from: https://www.scotlandscensus.gov.uk/marital-status-0 accessed 5 Jan 2021.

For peer review only

39. National Records of Scotland. Tenure of household 2018 [Available from: https://www.scotlandscensus.gov.uk/tenure-household accessed 5 Jan 2021.
40. Office for National Statistics. SOC 2000 [Available from: https://www.ons.gov.uk/methodology/classificationsandstandards/standardoccupationalclassificationsoc/socarchive accessed 5 Jan 2021.
41. The Scottish Government. Local government [Available from: https://www.gov.scot/policies/local-government/ accessed 5 Jan 2021.
42. The Scottish Government. Coronavirus (COVID-19): local protection levels 2021 [Available from: https://www.gov.scot/publications/coronavirus-covid-19-protection-levels/pages/overview/ accessed 5 Jan 2021.
43. The Scottish Government. Scottish Government Urban Rural Classification 2016. Edinburgh, 2018.
44. Stock SJ, McAllister D, Vasileiou E, et al. COVID-19 in Pregnancy in Scotland (COPS): protocol for an observational study using linked Scottish national data. *BMJ Open* 2020;10(11):e042813. doi: 10.1136/bmjopen-2020-042813 [published Online First: 2020/11/28]
45. Docherty AB, Harrison EM, Green CA, et al. Features of 20 133 UK patients in hospital with covid-19 using the ISARIC WHO Clinical Characterisation Protocol: prospective observational cohort study. *BMJ* 2020;369:m1985. doi: 10.1136/bmj.m1985 [published Online First: 2020/05/24]
46. Fine JP, Gray RJ. A Proportional Hazards Model for the Subdistribution of a Competing Risk. *Journal of the American Statistical Association* 1999;94(446):496-509. doi: 10.1080/01621459.1999.10474144
47. Naimi AI, Cole SR, Kennedy EH. An introduction to g methods. *Int J Epidemiol* 2017;46(2):756-62. doi: 10.1093/ije/dyw323 [published Online First: 2017/01/01]
48. Jackson JW, VanderWeele TJ. Intersectional decomposition analysis with differential exposure, effects, and construct. *Soc Sci Med* 2019;226:254-59. doi: 10.1016/j.socscimed.2019.01.033 [published Online First: 2019/02/17]
49. Knol MJ, VanderWeele TJ. Recommendations for presenting analyses of effect modification and interaction. *Int J Epidemiol* 2012;41(2):514-20. doi: 10.1093/ije/dyr218 [published Online First: 2012/01/19]
50. Lakha F, Gorman DR, Mateos P. Name analysis to classify populations by ethnicity in public health: validation of Onomap in Scotland. *Public Health* 2011;125(10):688-96. doi: 10.1016/j.puhe.2011.05.003 [published Online First: 2011/09/13]
51. Wilson N, Barnard LT, Summers JA, et al. Differential mortality rates by ethnicity in 3 influenza pandemics over a century, New Zealand. *Emerg Infect Dis* 2012;18(1):71-7. doi: 10.3201/eid1801.110035 [published Online First: 2012/01/20]

For peer review only

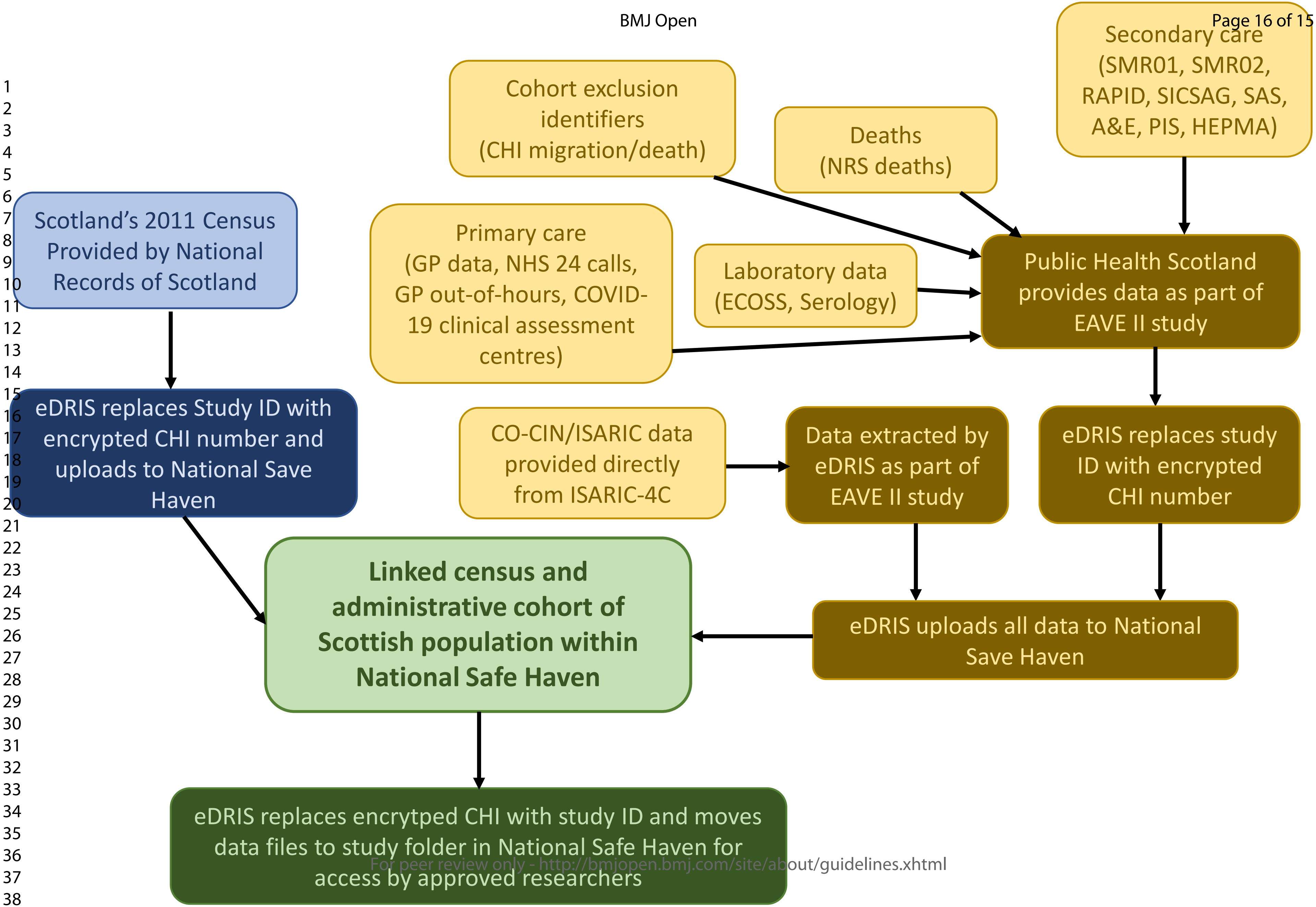

For peer review only - http://bmjopen.bmj.com/site/about/guidelines.xhtml

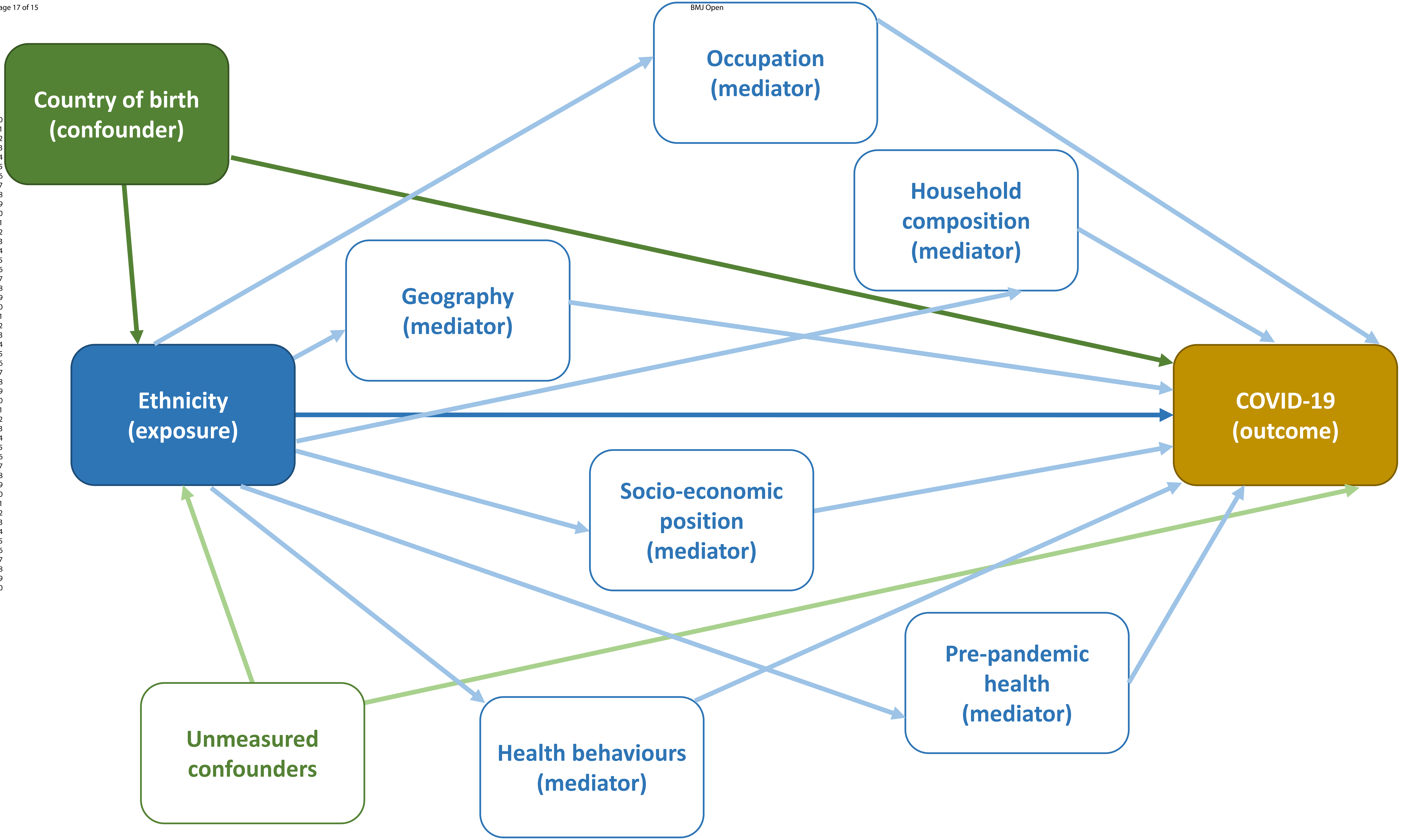
Country of birth (confounder)
Occupation (mediator)
Household composition (mediator)
Geography (mediator)
Ethnicity (exposure)
COVID-19 (outcome)
Socio-economic position (mediator)
Pre-pandemic health (mediator)
Unmeasured confounders
Health behaviours (mediator)
